# Plant NIGT1/HRS1/HHO Transcription Factors: Key Regulators with Multiple Roles in Plant Growth, Development, and Stress Responses

**DOI:** 10.3390/ijms22168685

**Published:** 2021-08-12

**Authors:** Qian Li, Luyan Zhou, Yuhong Li, Dongping Zhang, Yong Gao

**Affiliations:** 1 Jiangsu Key Laboratory of Crop Genomics and Molecular Breeding/Key Laboratory of Plant Functional Genomics of the Ministry of Education, Yangzhou University, Yangzhou 225009, China; LiQianyzu@163.com (Q.L.); zhouluyan1997@163.com (L.Z.); dpzhang@yzu.edu.cn (D.Z.); 2 Co-Innovation Center for Modern Production Technology of Grain Crops of Jiangsu Province, Joint International Research Laboratory of Agriculture and Agri-Product Safety of the Ministry of Education, Yangzhou University, Yangzhou 225009, China; 3 Institute of Agricultural Sciences for Lixiahe Region in Jiangsu, Yangzhou 225009, China; yhlirice@163.com

**Keywords:** NIGT1/HRS1/HHO transcription factors, G2-like, growth and development, abiotic stress, structure and function

## Abstract

The NIGT1/HRS1/HHO transcription factor (TF) family is a new subfamily of the G2-like TF family in the GARP superfamily and contains two conserved domains: the Myb-DNA binding domain and the hydrophobic and globular domain. Some studies showed that NIGT1/HRS1/HHO TFs are involved in coordinating the absorption and utilization of nitrogen and phosphorus. NIGT1/HRS1/HHO TFs also play an important role in plant growth and development and in the responses to abiotic stresses. This review focuses on recent advances in the structural characteristics of the NIGT1/HRS1/HHO TF family and discusses how the roles and functions of the NIGT1/HRS1/HHO TFs operate in terms of in plant growth, development, and stress responses.

## 1. Introduction

The GARP transcription factor (TF) superfamily is commonly found in plants. It is named for the Golden 2 (G2) protein in maize, the type B authentic response regulator (ARR-B) protein in *Arabidopsis thaliana*, and the phosphate starvation response 1 (PSR1) protein in *Chlamydomonas*. The GARP superfamily contains two types of TFs, ARR-B and G2-like TFs. Among them, the ARR-B TFs comprise the multi-functional domain B motif, which is related to nuclear localization and DNA binding. G2-like TFs have an Myb-DNA binding domain (Myb-DBD), which is highly similar to the B motif [1,2,3]. G2-like TFs are also known as Golden2-like and GLK and are derived from the G2 protein in maize [4,5]. G2-Like TFs have been found and characterized in monocots and dicots. Studies have shown that G2-Like TFs play an important role in chloroplast development, fruit quality, biotic and abiotic stress, plant senescence, and hormones.

NIGT1/HRS1/HHO TFs belong to a subfamily of the G2-Like TFs and were first identified and named in 2009 [6]. With advances in high-throughput sequencing technologies, more and more NIGT1/HRS1/HHO TF genes have been identified from *A. thaliana*, rice, and other species. Subsequently, the functions of NIGT1/HRS1/HHO TFs have been gradually found in nitrogen (N) and phosphorus (P) utilization, plant growth and development, and abiotic stress [7,8,9,10,11,12]. Here, the review focuses on recent advances in the structural characteristics and biological functions of the NIGT1/HRS1/HHO TFs.

## 2. Discovery and Naming of NIGT1/HRS1/HHO TF Family in Plants

**Figure 1:**
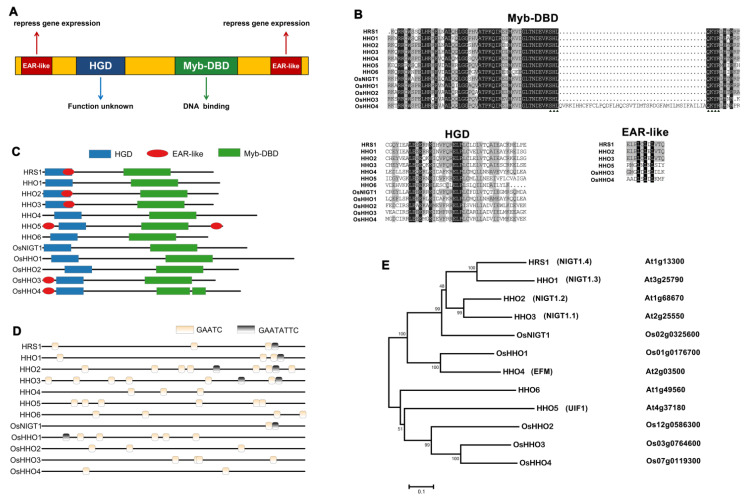
Classification and structure of the NIGT1/HRS1/HHO family. (**A**) The structural model of the NIGT1/HRS1/HHO family consisting of three types of conserved domains: Myb-DBD (green), HGD (blue), and EAR like (red). (**B**) The sequence alignment of the conserved domains of the NIGT1/HRS1/HHO family. Fully conserved amino acids are shaded in black, and similar amino acids (>50% identical) are shaded in gray. At the top, the conserved Myb-DBD domain contains an HLH motif and an SHLQKYR sequence tagged with a triangle. There is the conserved C-terminal hydrophobic domain HGD at the bottom left. The bottom right partially contains an EAR-like motif (LxLxL, where L is leucine, and X is any amino acid). (**C**) A schematic diagram showing the domains of various NIGT1/HRS1/HHO TFs in *A. thaliana* and rice. (**D**) There are two types of cis-regulatory motifs, GAATC and GAATATTC, in the 2500 bp promoter region of various NIGT1/HRS1/HHO TFs in *A. thaliana* and rice. (**E**) The phylogenetic tree of the NIGT1/HRS1/HHO family constructed on the basis of the neighbor-joining method, and the gene IDs and names of each gene are displayed.

In 2009, Liu et al. [6] first reported that the G2-like TF gene *At1g13300* named *HRS1* exhibited hypersensitivity to the shortening of primary roots induced by low phosphate (Pi) in *A. thaliana*. Further study found that *HRS1* has six homologous genes in the *A. thaliana* (i.e., *At1g25550*, *At1g49560*, *At1g68670*, *At2g03500*, *At3g25790*, and *At4g37180*), and these genes were named as *HHO1* (*HRS1 Homolog 1*) to *HHO6*. This family was then named the HRS1/HHO TF family. In 2013, a nitrate-induced GARP transcription repressor gene *NIGT1* (*Os02g0325600*) was reported to belong to the HRS1/HHO family in rice. A total of four homologous genes of *NIGT1* (i.e., *Os01g0176700*, *Os03g0764600*, *Os07g0119300*, and *Os12g0586300*) were also found in rice. Meanwhile, the study showed that *HRS1*, *HHO1*, *HHO2*, and *HHO3* in *A. thaliana* had homology to *NIGT1* in rice, and all of their expressions were strongly induced by nitrate [7]. Subsequently, *HRS1* and *HHO1*–*HHO5* were also named *NIGT1.4*, *NIGT1.3*, *NIGT1.2*, *NIGT1.1*, *EFM*, and *UIF1*, respectively [8,9,10]. Recently, four *NIGT1* homologous genes were also named *OsHHO1*–*OsHHO4* in rice [11]. Since then, there are two naming methods for the HRS1/HHO family in *A. thaliana* and rice (Figure 1). The genes of the HRS1/HHO TF family have been found in other species. The HRS1/HHO family genes, *VaAQUILO* (*VaAQ*) in *V. amurensis* and *VvAQUILO* (*VvAQ*) in *V. vinifera*, were first reported in 2018 [12]. However, other homologous genes of *VaAQ* and *VvAQ* have not been found in *V. amurensis* and *V. vinifera*. In summary, in accordance with the naming and unity principles, seven members of this family were named *HRS1* and *HHO1*–*HHO6* in *A. thaliana*, and five members were named *OsNIGT1* and *OsHHO1*–*OsHHO4* in rice. Finally, this family was called the NIGT1/HRS1/HHO TF family.

## 3. Structure of NIGT1/HRS1/HHO TF Family

NIGT1/HRS1/HHO TFs belong to a subfamily of G2-like TFs. All G2-like TFs have highly conserved Myb-DBD, and this domain contains an HLH (Helix–Loop–Helix) motif. The HLH motif can bind to DNA to regulate various physiological processes and can participate in the dimerization of transcriptional regulators. In an HLH motif, the first helix consists of 14 amino acids starting from PELHRR and its variant motif. The second helix starts from NI/VASHLQ and its variant motifs and extends to different lengths in various G2-Like proteins. Between the two helices is a loop containing 22 amino acids [13,14]. The C terminal of Myb-DBD contains a conserved SHLQ(K/M) (Y/F) R motif [3]. Studies have also shown that some G2-like proteins have a GLK/C-terminal box (GCT box) located in the C-terminal part. This GCT box is encoded by the last exon and plays a role in the dimerization process [14] (Figure 1A,B).

In addition to Myb-DBD, NIGT1/HRS1/HHO TFs also have a conserved hydrophobic and globular domain (HGD), which is rich in hydrophobic amino acids at the N terminal. Up until now, the function of HGD has not yet been studied. Moreover, some NIGT1/HRS1/HHO TFs also contain one or two different EAR-like motifs at their N or C terminal [9]. EAR-like motifs play an important role in inhibiting gene expression as transcription repressors or recruit corepressors [15,16] (Figure 1A). We analyzed the conserved domains of 12 NIGT1/HRS1/HHO family genes in rice and *A. thaliana*. All NIGT1/HRS1/HHO proteins contain two conserved domains: Myb-DBD (C-terminal contains conserved SHLQKYR motif) and an HGD domain. HRS1, HHO2, HHO3, OsHHO3, and OsHHO4 have an EAR-like motif at the N terminal. HHO5 has two different EAR-like motifs at the N and C terminals (Figure 1B,C).

OsNIGT1 was found to be a transcribed inhibitory factor and could repress the transcription of downstream genes and itself. Further research showed that OsNIGT1 could bind to GAATC as a monomer or through a subunit of the dimer. OsNIGT1 also could bind to GAATATTC through the interaction of two subunits of the dimer. These two binding sites were also found in the promoters of some NIGT1/HRS1/HHO TFs genes in *A. thaliana* [7,17]. Therefore, the autorepression mechanism of OsNIGT1 may be conserved in monocotyledons and dicotyledons. This review further analyzed the binding sites of the promoter of all NIGT1/HRS1/HHO family genes in *A. thaliana* and rice (Figure 1D). The result indicated that the promoters of all NIGT1/HRS1/HHO family genes have the binding sites for autorepression in *A. thaliana* and rice. It is suggested that the NIGT1/HRS1/HHO TF family might have the autorepression mechanism universally.

## 4. Function of NIGT1/HRS1/HHO TF Family the Absorption and Utilization of Nitrogen and Phosphorus in Plant

Nitrogen (N) and phosphorus (P) are the key elements for plant growth and exist in many forms in nature. In organisms, P appears in the form of free phosphate ions, which are called inorganic phosphate (Pi). Because of the limited Pi content in the soil, plants are often in a Pi deficient state, which leads to a series of Pi starvation responses (PSR) [18,19]. The studies on the absorption and utilization of P by NIGT1/HRS1/HHO TFs are mainly concentrated in *A. thaliana*. Under Pi deficiency, the expression of *HRS1* and *HHO1* were increased in leaves and roots, suggesting that *HRS1* and *HHO1* played a role in PSR [6,20]. In addition, HHO2 can regulate its Pi homeostasis by Phosphate Starvation Response 1 protein (PHR1), which is the major regulator of PSR [21].

Nitrate is the main N source and the N signaling source for most land plants [7,22]. Some studies found that NIGT1/HRS1/HHO TFs arealso regulators of N starvation responses in *A. thaliana* and played an important role in N absorption and utilization. Under N starvation, the expression of NIGT1/HRS1/HHO TFs (i.e., *HRS1*, *HHO1*, *HHO2*, and *HHO3*) decrease rapidly, and the expression of N starvation response genes increase, which leads to N starvation responses. After N application, the expression of NIGT1/HRS1/HHO TFs (i.e., *HRS1*, *HHO1*, *HHO2*, and *HHO3*) is rapidly induced to repress the expression of N starvation gene responses, which reduce N starvation responses [23]. In addition, *HRS1* and *HHO1* were found to be the early induced genes and major participants in nitrate signaling. The expression of *HRS1* and *HHO1* is regulated by the nitrate response transcription factors NIN-LIKE PROTEIN (NLP) and the nitrate transporters NRT1.1/NPF6.3 and NRT2.1 [10,24,25,26]. NIGT1/HRS1/HHO TFs also represses the expression of other genes related to N utilization, such as *NITRATE REDUCTASE 1* (*NIA1*), *GLUTAMINE SYNTHE-TASE 1;1* (*GLN1;1*) and *GLN1;4*. It is suggested that NIGT1/HRS1/HHO TFs can prevent the excessive accumulation of N by transducing the nitrogen saturation signal [27].

**Figure 2:**
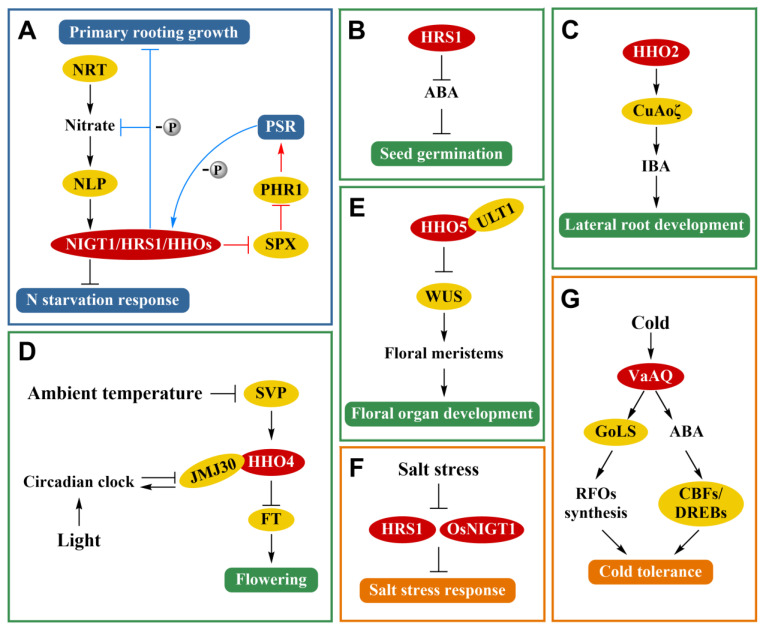
Simplified schematic of NIGT1/HRS1/HHO TFs involved in multifunctional regulation in plants. (**A**) The black arrow: Nitrate transporter NRT can increase the content of nitrate and can enhance the expression of the nitrate response transcription factors and induce the expression of NIGT1/HRS1/HHO family genes to inhibit the N starvation response. The red arrow: NIGT1/HRS1/HHO TFs can inhibit the expression of SPX and can induce the expression of *PHR1* to promote PSR. The blue arrow: Under Pi deficiency, PSR can promote the expression of NIGT1/HRS1/HHO TFs to inhibit nitrate absorption and primary root growth. (**B**–**E**) Functions of NIGT1/HRS1/HHO TFs in plant growth and development. (**B**) HRS1 can promote seed germination by inhibiting ABA signaling. (**C**) HHO2 can increase the expression of *CuAOζ* and promotes IBA to enhance lateral root growth. (**D**) NIGT1/HRS1/HHO TFs are involved in flowering by coordinating temperature and light. SVP can promote the expression of *HHO4*. HHO4 can interact with JMJ30. Protein complex of HHO4 and JMJ30 repress the expression of *FT* gene to inhibit flowering. (**E**) HHO5 can interact with ULT1 and can inhibit the expression of *WUS* to participate in the homeostasis of floral meristems and the growth and development of floral organs. (**F**,**G**) Functions of NIGT1/HRS1/HHO TFs in abiotic stress. (**F**) The expression of *HRS1* and *OsNIGT1* are repressed under salt stress. Mutants of *HRS1* and *OsNIGT1* can enhance tolerance to salt stress. (**G**) VaAQ can improve low temperature tolerance. VaAQ increased the synthesis of RFOs by increasing GoLS activity to improve low temperature tolerance. VaAQ also regulates the expression of the *CBFs* or *DREBs* and improves tolerance to low temperature stress through the ABA pathway. Promoting and repressive effects are indicated by → and ┤, respectively, and P deficiency is denoted by -P.

The balance of nitrogen and phosphorus is essential for the normal growth and development of plants. Studies have demonstrated that PHR1 can promote the expression of *HRS1* by modulating PSR and reducing nitrate absorption. HRS1 and HHO1 can coordinate N and P by regulating the nitrate and phosphate starvation responses in *A. thaliana* [10,26,28,29]. Further study showed that HRS1, PHR1, and SPX (SYG1/PHO81/XPR1, the inhibitory factor of PHR1) could form the NIGT1-SPX-PHR signaling pathway, which regulates the N content and coordinates the balance of N and P in response to PSR [10,23,29]. Moreover, the study showed that HHO5 and HHO6 were related to N and P responses and were the regulators of N absorption [30] (Figure 2A). 

Similar to NIGT1/HRS1/HHO family genes in *A. thaliana*, the expression of *OsNIGT1* can be rapidly and specifically induced by nitrate in rice. OsNIGT1 can also regulate nitrate utilization and can play critical roles in nitrate signaling in rice [7]. OsHHO3 and OsHHO4 are the key regulators in response to N deficiency and may play a role as the transcriptional inhibitors in N utilization [11,31]. However, the P absorption and utilization functions of NIGT1/HRS1/HHO TFs have yet to be reported in rice. In summary, NIGT1/HRS1/HHO TFs can regulate gene expression in the nitrate and phosphate signaling pathways and can coordinate nitrate and phosphate absorption. It is suggested that NIGT1/HRS1/HHO TFs play an important role in balancing the absorption and utilization of N and P in plants.

## 5. Functions of NIGT1/HRS1/HHO TF Family in Plant Growth and Development

Evidence suggests that NIGT1/HRS1/HHO TFs not only participate in the absorption and utilization of N and P, but also show complex regulatory mechanisms and networks involved in seed germination, root development, and flowering. An overexpression of *HRS1* could inhibit the elongation of primary roots in the absence of Pi. HHO1 could also inhibit the growth of primary roots under a specific ratio of N and P. The double knockout mutants of *HRS1* and *HHO1* significantly inhibit the growth of primary roots under nitrate only treatment [6,28] (Figure 2A). In addition, HRS1 is a negative regulator of ABA signaling and can promote seed germination by inhibiting ABA signaling in *A. thaliana*. HRS1 can enhance H^+^-ATPase activity on the cell membrane and the elongation of cells in the hypocotyl and hypocotyl–radicle transition zone to promote seed germination in *A. thaliana* [32] (Figure 2B). In addition to being related to primary roots growth, NIGT1/HRS1/HHO TFs can also promote the development of lateral roots. The copper amine oxidase ζ (CuAOζ) is located in peroxisomes in *A. thaliana*. CuAOζ-derived ROS is involved in the development of lateral roots induced by indole-3-butyric acid (IBA) [33]. HHO2 can increase the expression of *CuAOζ* and can enhance lateral root growth [21,34] (Figure 2C).

A recent study showed that HHO4 and HHO5 were associated with flowering. The short vegetative phase (SVP) TF is affected by temperature to regulate flowering. SVP can promote the expression of *HHO4* in *A. thaliana*. Meanwhile, HHO4 can interact with JMJ30, which is the H3K36Me2 demethylase and is involved in light-responsive circadian clock. The protein complexes of HHO4 and JMJ30 repress the expression of the *FLOWERING LOCUS T* (*FT*) gene to inhibit flowering. It is suggested that NIGT1/HRS1/HHO TFs are involved in flowering by coordinating temperature and light [8] (Figure 2D). Ultrapetala1 (ULT1) is a key negative regulator of stem cell activity in shoot apical meristems and floral meristems. HHO5 can interact with ULT1 and the inhibited expression of the central transcription factor WUSCHEL (WUS), which controls the balance of shoot apical meristems in apical meristems. HHO5 and ULT1 can participate in the homeostasis of floral meristems and the growth and development of floral organs [9] (Figure 2E). In conclusion, NIGT1/HRS1/HHO TFs may be involved in seed germination, root development, and flowering by responding to light, temperature, and hormone (ABA and IBA) signals. Therefore, NIGT1/HRS1/HHO TFs play an important role in plant growth and development.

## 6. Function of NIGT1/HRS1/HHO TF Family in Plant Abiotic Stress

Up until now, few studies have been reported on the relationship between NIGT1/HRS1/HHO TFs and abiotic stress. Some studies have found that NIGT1/HRS1/HHO TFs may be related to salt and low temperature stress. The expression of *HRS1* was repressed under salt stress in *A. thaliana* [35]. Further research has demonstrated that the membrane ion leakages of the *HRS1* mutant and *OsNIGT1* mutant significantly decreases in *A. thaliana* and rice, respectively, and the *HRS1* mutant and *OsNIGT1* mutant could continue to grow and fruit in *A. thaliana* and rice after salt stress. It is suggested that the NIGT1/HRS1/HHO TFs are related to salt tolerance [35] (Figure 2F). *VaAQ*, a grape TF gene homologous to *HRS1*, *HHO2*, and *HHO3*, can improve the low temperature tolerance in transgenic *A. thaliana* and transgenic *V. amurensis*. VaAQ increases the synthesis of raffinose family oligosaccharides (RFOs) by increasing the activity of galactinol synthase (GoLS) to improve low temperature tolerance. Through the ABA pathway, VaAQ also regulates the expression of *CBFs* or *DREBs,* which are the core genes of low-temperature signaling, and improves tolerance to low temperature stress [12] (Figure 2G).

## 7. Conclusions

The NIGT1/HRS1/HHO TF family is a new family that was first named in 2009. The NIGT1/HRS1/HHO TF family has seven members (HRS1 and HHO1–HHO6) in *A. thaliana* and five members (OsNIGT1 and OsHHO1–OsHHO4) in rice. NIGT1/HRS1/HHO TFs play an important coordinating and regulating role of N and P absorption and utilization, growth and development, and abiotic stress. The coordinated regulation mechanism of plant nutrition and development is essential to cope with the ever-changing environment [3]. Roots play a key role in the adaptive responses mediated by Pi deficiency. Meanwhile, Pi affects the growth of primary roots [36,37]. Nitrates are related to the growth of lateral roots and can counteract the effect of glutamate on the growth of primary roots [38]. However, the mechanism by which plants integrate these key nutrients to regulate development is still unknown. NIGT1/HRS1/HHO TFs have been shown to coordinate the absorption and utilization of N and P and to participate in root development regulated by N and P through unknown mechanisms. Therefore, NIGT1/HRS1/HHO TFs are the key to understanding the relationship between the N and P signaling pathways and root development. In addition, previous studies on NIGT1/HRS1/HHO TFs have mainly focused on the utilization of N and P. The role of NIGT1/HRS1/HHO TFs in plant growth and abiotic stress has not been studied in depth. Currently, NIGT1/HRS1/HHO TFs have been found to regulate seed germination and to enhance low-temperature stress tolerance through the ABA signaling pathway. These studies also provide references for further research on the molecular mechanism between NIGT1/HRS1/HHO TFs and ABA signals. Moreover, whether NIGT1/HRS1/HHO TFs are also involved in the synthesis or signaling pathways of other plant hormones remains to be further explored. In conclusion, although many questions remain unanswered, further research will expand our understanding of the function of NIGT1/HRS1/HHO TFs in plant growth, development, and stress responses.

## Data Availability

Not applicable.
